# Evaluation of 11 SARS-CoV-2 antibody tests by using samples from patients with defined IgG antibody titers

**DOI:** 10.1038/s41598-021-87289-6

**Published:** 2021-04-07

**Authors:** Nina Lagerqvist, Kimia T. Maleki, Jenny Verner-Carlsson, Mikaela Olausson, Joakim Dillner, Julia Wigren Byström, Tor Monsen, Mattias Forsell, Jenny Eriksson, Gordana Bogdanovic, Sandra Muschiol, Joel Ljunggren, Johanna Repo, Torbjörn Kjerstadius, Shaman Muradrasoli, Mia Brytting, Åsa Szekely Björndal, Thomas Åkerlund, Charlotta Nilsson, Jonas Klingström

**Affiliations:** 1 Department of Microbiology, Public Health Agency of Sweden, Solna, Sweden; 2 Department of Medicine Huddinge, Karolinska Institutet, Stockholm, Sweden; 3 Department of Laboratory Medicine, Karolinska Institutet, Stockholm, Sweden; 4 Department of Clinical Microbiology, Umeå University, Umeå, Sweden; 5 Region Västmanland, Västerås, Sweden; 6 Karolinska University Hospital, Stockholm, Sweden; 7 Region Västernorrland, County Hospital of Västernorrland, Sundsvall, Sweden; 8 Region Värmland, Centralsjukhuset, Karlstad, Sweden

**Keywords:** Immunology, Microbiology

## Abstract

We evaluated the performance of 11 SARS-CoV-2 antibody tests using a reference set of heat-inactivated samples from 278 unexposed persons and 258 COVID-19 patients, some of whom contributed serial samples. The reference set included samples with a variation in SARS-CoV-2 IgG antibody titers, as determined by an in-house immunofluorescence assay (IFA). The five evaluated rapid diagnostic tests had a specificity of 99.0% and a sensitivity that ranged from 56.3 to 81.6% and decreased with low IFA IgG titers. The specificity was > 99% for five out of six platform-based tests, and when assessed using samples collected ≥ 22 days after symptom onset, two assays had a sensitivity of > 96%. These two assays also detected samples with low IFA titers more frequently than the other assays. In conclusion, the evaluated antibody tests showed a heterogeneity in their performances and only a few tests performed well with samples having low IFA IgG titers, an important aspect for diagnostics and epidemiological investigations.

## Introduction

Severe acute respiratory syndrome coronavirus 2 (SARS‐CoV‐2) was identified in January 2020 and has since spread globally reaching pandemic proportions. The clinical picture of COVID-19 ranges from asymptomatic persons to patients presenting various symptoms with mild or severe disease^1^. Given the acute onset of COVID-19, nucleic acid amplification tests (NAATs) play an important role in diagnostics of patients^2^. NAATs generally show high sensitivity and specificity, but the false-negative rate can be high depending on when in the disease course they are used^3^. Unlike NAATs, antibody tests allow for diagnosis of recent and past infections. The potential role of IgM and IgG as markers for COVID-19 has been evaluated^4–9^. IgM antibodies can become detectable during the first week of illness and a majority of patients develops IgM antibodies by week two after onset of symptoms^4–7^. Similarly, IgG antibodies toward different SARS-CoV-2 antigens first become detectable during the first week^10^ and by the third week, > 90% of patients with mild or severe COVID-19 have detectable IgG antibodies^5,8,9^.

Several in-house and commercial antibody tests have been produced based on recombinant nucleocapsid (N), spike (S), S1 subunit, or receptor binding domain (RBD) SARS-CoV-2 antigens^11–13^. Antibody tests need to have high sensitivity and specificity to be valuable in diagnostics and to enable contact tracing and support surveillance efforts. This is particularly important as studies suggest that persons with previous asymptomatic or mild SARS-CoV-2 infections may have a weaker antibody response to SARS-CoV-2 than moderately to severely ill patients^14–16^. Moreover, the current knowledge regarding long-term antibody responses is limited, but similar to other acute viral infections there are reports of waning antibody levels over time^17–20^. To our knowledge, few studies have addressed how antibody levels impact the performance of SARS-CoV-2 antibody tests.

We evaluated the performance of five rapid diagnostic tests (RDTs) and six platform-based assays using 306 samples from patients with laboratory confirmed COVID-19 and 278 samples from persons with no previous history of SARS-CoV-2 infection. As most available antibody tests are qualitative or semi-qualitative in design, we determined the anti-SARS-CoV-2 IgG antibody titer in all samples from COVID-19 patients using an in-house immunofluorescence assay (IFA), thereby allowing comparison of assay-performance in samples with defined IgG antibody titers.

## Material and methods

### Samples

**Figure 1:**
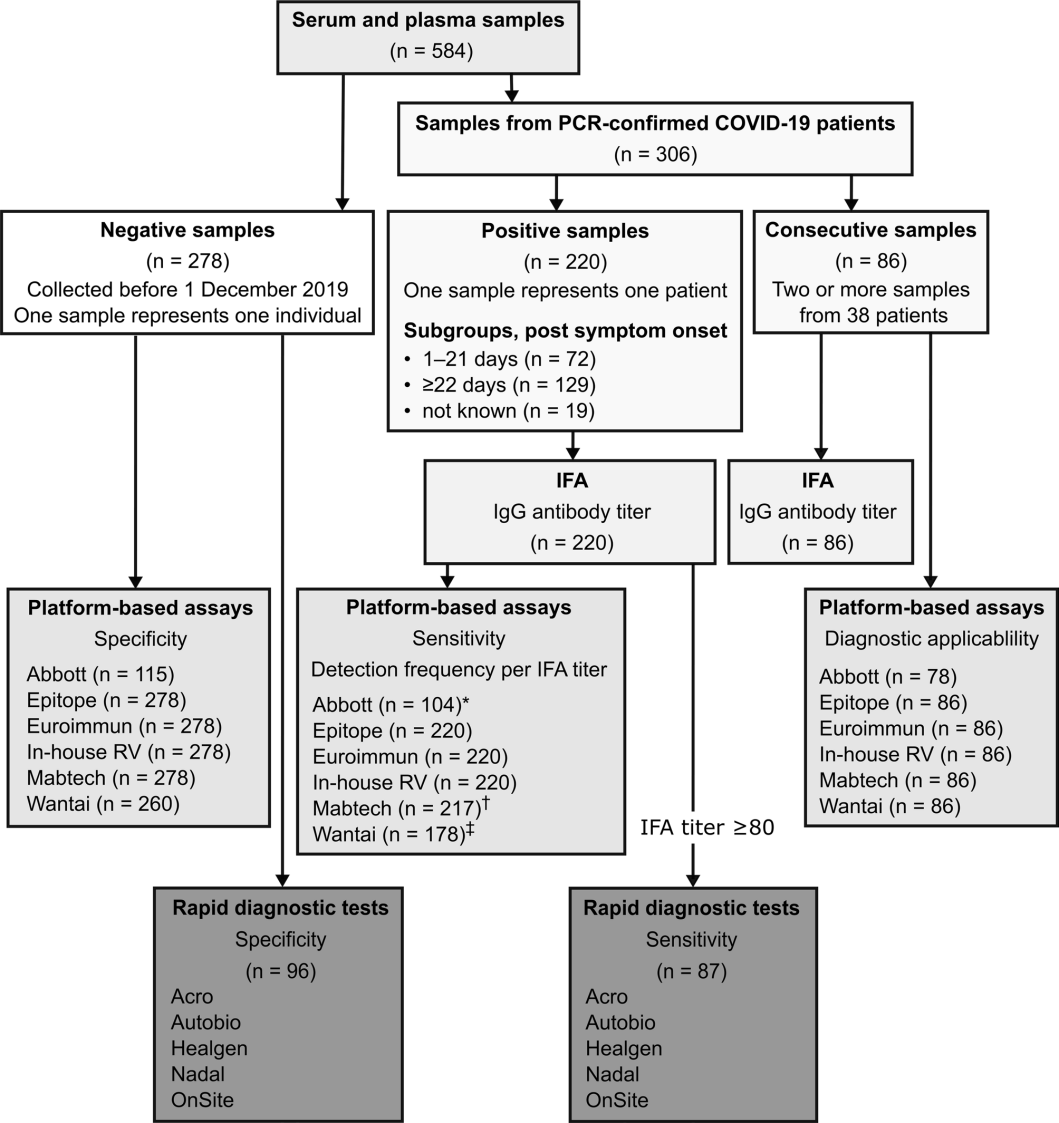
Schematic overview of samples and antibody detection tests.* IFA* immunofluorescence assay. *32/72 samples collected 1–21 days post symptom onset, 65/129 samples collected ≥ 22 days post symptom onset, and 7/19 samples lacking information regarding elapsed time between symptom onset and sampling.^†^126/129 samples collected ≥ 22 days post symptom onset.^‡^15/72 samples collected 1–21 days and 94/129 samples collected ≥ 22 days post symptom onset.

The samples used to assess the performance of the antibody tests were 278 serum or plasma samples from SARS-CoV-2 seronegative persons, 220 serum samples from COVID-19 patients, and 86 samples from 38 COVID-19 patients who were sampled at least twice after symptom onset (Fig. 1). The diagnosis of all COVID-19 patients was confirmed by NAAT.

The 278 negative samples were collected before 1 December 2019 and were from 35 healthy donors, 164 persons seeking medical care, and 79 patients with infectious diseases, out of which 32 were caused by bacteria, 7 by parasites, and 40 were caused by viruses (Supplementary Text). The latter set of samples included 16 samples from patients infected by human coronaviruses: NL63 (n = 6) and 229E (n = 3), and patients co-infected with OC43 and HKU1 (n = 7). Of the 278 negative sample donors, 102 (37%) were men and 132 (47%) were women, and information on sex was missing for 44 persons (16%). The median age was 31 years (range 2–83 years).

The 220 COVID-19 positive samples (each sample representing one person) were categorized into two subsets based on the time interval between symptom onset and sampling: 1–21 and ≥ 22 days post-symptom onset. A third subset consisted of samples for which information on symptom onset, sampling date, or both were missing. With few exceptions, the samples were collected within 3 months post-symptom onset. Of the 220 positive sample donors 54 (25%) were men and 41 (19%) were women, and information on sex was missing for 125 (57%) patients. The median age of the COVID-19 patients was 54 years (range 15–90 years). For 63 patients, information on disease severity was available; 20 were outpatients and 43 were hospitalized.

The 86 consecutively collected samples were categorized into seven subsets: samples collected week 1, 2, 3, 4, 5, 6, and week 7 after symptom onset. The samples were stored at – 20 °C pending analysis and working aliquots at + 4 °C.

Studies have demonstrated a loss in infectivity of coronaviruses after heating^21^. We implemented a precautionary safety protocol and all samples were subjected to heat treatment at 56 °C for 30 min before use. Initial testing revealed that one RDT had difficulties processing heat-inactivated samples. For this reason, heat-inactivated samples were subjected to a short centrifugation (1 min at 1000×*g*) before being applied to RDT cassettes. Centrifugation markedly reduced the number of invalid tests and was adopted for testing of RDTs.

### Immunofluorescence assay

The anti-SARS-CoV-2 IgG antibody titer was determined for all samples from COVID-19 patients by using an in-house IFA as previously described^22^. Briefly, Vero E6 cells (ATCC CRL-1586) infected with SARS-CoV-2 (GenBank accession no. MT093571) were seeded on microscope slides and then fixed. Samples were tested at two-fold serum dilutions and bound anti-SARS-CoV-2 IgG antibody was visualized using AF488-conjugated AffiniPure goat anti-human IgG antibody (Jackson Immunoresearch) in a Nikon Eclipse Ni fluorescence microscope. The IFA was evaluated by using samples from 45 persons with no prior history of COVID-19 and all samples were found negative in IFA at a dilution of 1:40. We therefore decided to analyze samples from COVID-19 patients in two-fold dilutions starting at a dilution of 1:80. The intensity of the fluorescence was determined independently (blinded reading) by two laboratory technicians experienced in both performing and interpreting IFA results and was graded as none (no or unspecific fluorescence) or present (rated “+”, “++”, or “+++”). The titer was expressed as the reciprocal of the highest dilution resulting in “+”. No fluorescence at dilution 1:80 are referred to as a titer < 80. A fluorescence intensity of “++” or “+++” at a dilution of 1:320 was referred to as a titer of > 320.

### Rapid diagnostic tests

Five RDTs were included in the evaluation; 2019-nCoV IgG/IgM Rapid Test (Acro Biotech Inc., Rancho Cucamonga, CA, USA), Anti-SARS-CoV-2 Rapid Test (Autobio Diagnostics Co. Ltd, Zhengzhou, China), COVID-19 IgG/IgM Rapid Test (Healgen Scientific Limited Liability Company, Houston, TX, USA/Zhejiang Orient Gene Biotech Co. Ltd, Zhejiang, China), NADAL COVID-19 IgG/IgM Test (Nal von Minden GmbH, Moers, Germany), and OnSite COVID-19 IgG/IgM Rapid Test (CTK Biotech Inc., Poway, CA, USA). The RDTs are referred to as Acro, Autobio, Healgen, Nadal, and OnSite and their IgG results are presented here. The characteristics of the RDTs are summarized in Table S1. Because of limited sample volume, their performances were evaluated using a subset of the panel, which included 96 negative and 87 positive samples. The criteria for inclusion of positive samples was an IFA IgG titer of ≥ 80.

The RDTs were performed according to the manufacturers’ instructions. A test result was classified as negative (no detectable band), positive (clear band), or inconclusive (shade of colored line in the test-line-region) independently by two laboratory technicians using blinded reading. Discordant results between the technicians were handled as follows; an inconclusive and a positive result were interpreted as a positive result, and an inconclusive and a negative result were interpreted as an inconclusive result.

### Platform-based tests

Six platform-based tests were evaluated; Architect SARS-CoV-2 IgG (Abbott, Chicago, IL, USA), EDI Novel coronavirus COVID-19 IgG ELISA (Epitope Diagnostics Inc., San Diego, CA, USA), Anti-SARS-CoV-2 ELISA (IgG) (Euroimmun, Lübeck, Germany), SARS-CoV-2 IgG S-ELISA (in-house Region Västerbotten (in-house RV))^23^, SARS-CoV-2 Spike S1-RBD Ig Bridge ELISA (MabTech AB, Stockholm, Sweden), and Wantai SARS-CoV-2 Ab ELISA (Beijing Wantai Biological Pharmacy Enterprise Co., Ltd., Beijing, China). The assays are here referred to as Abbott, Epitope, Euroimmun, in-house RV, Mabtech, and Wantai and their characteristics are shown in Table S1.

Commercial antibody tests were performed according to the manufacturers’ instructions with the exception of the use of heat-inactivated samples. For all commercial tests, the cutoff was calculated according to the package insert. In-house RV was used with a cutoff of 0.7^23^. Samples were tested in duplicates in Euroimmun, Epitope, and in-house RV and the average absorbance reading of each duplicate was used for subsequent calculations according to the manufacturers’ instructions. Samples were tested in single wells in Abbott, Mabtech, and Wantai as they required an input volume of 300 µL, 25 µL, and 100 µL, respectively. Epitope, Euroimmun, and In-house RV were evaluated using the full set of negative and positive samples and Abbott, Mabtech, and Wantai with a subset of the samples. Samples were only tested once if not stated otherwise. The difference in the number of samples used and the number of replicates per assay were due to the available sample volume.

### Statistical analysis

For the calculation of sensitivity and specificity, an inconclusive RDT result for a known negative sample was considered a positive result, while a known positive sample (IFA titer ≥ 80) with an inconclusive RDT was classified as a negative result. A borderline outcome in a platform-based test were considered as a negative result. Sensitivities and specificities with Wilson-score 95% confidence interval (CI) and interrater agreement (Cohen’s kappa, κ) with standard error (SE) were calculated by using STATA version 15.1. Wilcoxon matched-pairs signed rank test was performed and graphs were made by using GraphPad Prism version 8.

### Ethical statement

The evaluation was performed as part of the Public Health Agency of Sweden’s assignment to monitor communicable diseases, evaluate infection control measures, and support laboratories in diagnostic development and quality assessments in accordance with §§ 3.6 and 3.8 of the ordinance (2013, p. 1020) from the Swedish Parliament. The leftover samples used in the evaluation were either obtained from the biobank repository at the Public Health Agency of Sweden or provided by clinical laboratories. These samples were used as stipulated in the regulations for use of such material in diagnostic quality assessment.

## Results

We selected five RDTs and four platform-based tests based on the presence of European Conformity (CE) marking and kit availability at the time of the evaluation (April to July 2020). One commercial ELISA (Mabtech) without CE marking and one in-house ELISA (in-house RV), primarily used for tracing of asymptomatic contacts in one of Sweden’s 21 regions^23^, were also included in the evaluation. In addition, the anti-SARS-CoV-2 IgG antibody titer was determined for all samples from COVID-19 patients using an in-house IFA.

### Performance of rapid diagnostic tests

#### Inconclusive results

RDTs provide a qualitative result (positive/negative), but we sometimes noted a diffuse line appearing in the test-line-region, here defined as an inconclusive result. The frequency of inconclusive IgG results ranged from 3.4% (3/87) to 12.5% (11/87) depending on the RDT applied (Table S2). Diffuse lines can make interpretation difficult, but despite that, the level of agreement between the two laboratory technicians’ interpretations was high (98.9%, κ = 0.978, SE = 0.029) (Table S3).

#### Specificity and sensitivity

**Figure 2:**
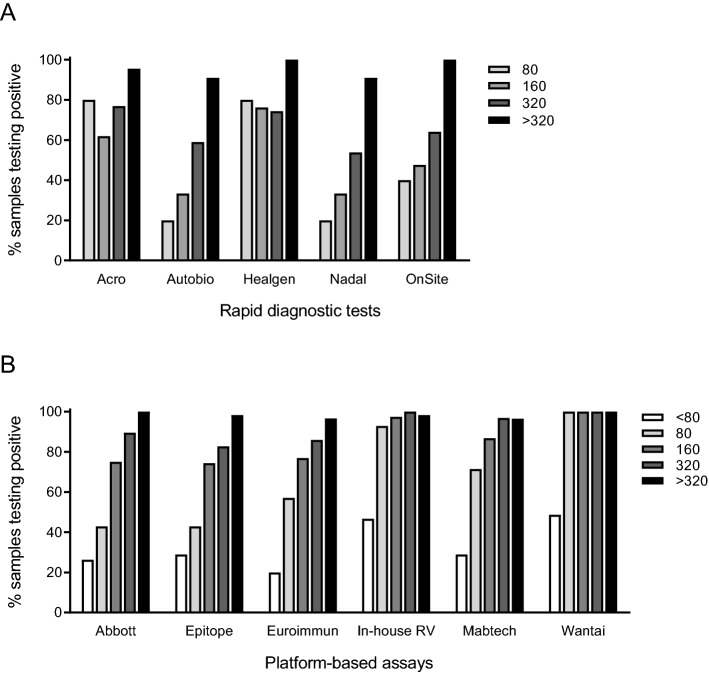
Performance of antibody tests assessed using samples with defined IgG antibody titers. The IgG titers, ranging from 80 to > 320, were determined using an in-house immunofluorescence assay (IFA). (**A**) The proportion of samples testing positive in rapid diagnostic IgG tests. The reference set of positive samples included 5 samples with a IgG titer of 80, 21 samples with a titer of 160, 39 samples with a titer of 320, and 22 samples with a IFA titer of > 320. (**B**) The proportion of samples testing positive in platform-based assays. Epitope, Euroimmun, and in-house RV were evaluated using 220 positive samples and Abbot, Mabtech, and Wantai were evaluated using 104, 217, and 178 positive reference samples, respectively. Among the tested reference samples, 45 tested negative at a dilution of 1:80 (< 80) using IFA, 14 samples had a titer of 80, 39 samples had a titer of 160, 64 samples had a titer of 320, and 58 samples had an IFA titer of > 320.

The specificity was 99.0% (95% CI 94.3–100) for all RDTs and the sensitivity ranged from 56.3 to 81.6%, and increased with increasing IFA titer (Table 1, Fig. 2A). Though partly overlapping CI with other tests, Acro and Healgen had the highest sensitivity of the five evaluated RDTs, 78.2% (95% CI 68.0–86.3) and 81.6% (95% CI 71.9–89.1), respectively (Table 1). Acro and Healgen were also the RDTs that most frequently detected samples with IFA titers 80 and 160 (Fig. 2A).

**Table 1:** Specificity and sensitivity of five SARS-CoV-2 rapid diagnostic antibody tests.

Rapid diagnostic test	Specificity (96 negative samples)		Sensitivity (87 positive samples)	
	%	95% CI	%	95% CI
Acro	99.0	94.3–100	78.2	68.0–86.3
Autobio	99.0	94.3–100	58.6	47.6–69.1
Healgen	99.0	94.3–100	81.6	71.9–89.1
Nadal	99.0	94.3–100	56.3	45.3–66.9
OnSite	99.0	94.3–100	67.8	56.9–77.4

*CI* confidence interval.

### Performance of platform-based tests

#### Effect of heat treatment of samples

To investigate possible effects of heat treatment on assay performance, non-heat-inactivated and heat-inactivated aliquots of 27 samples (6 negative and 21 positive samples) were tested in parallel in Euroimmun, Epitope, Mabtech, and Wantai, and 6 positive samples were tested in Abbott. No differences in the obtained results (positive/negative) were observed for Abbott, Wantai, and Mabtech (Fig. S1). Using Euroimmun, one positive sample tested positive when non-heat-inactivated and borderline when heat-inactivated. Using Epitope, the non-heat-inactivated aliquot of one negative sample tested borderline but tested negative when heat-inactivated.

Significant differences (p < 0.0001 and p = 0.0245) between optical density ratios of non-heat-inactivated and heat-inactivated samples were observed for Euroimmun and Wantai, whereas only minor differences were observed for Abbott, Epitope, and Mabtech (Fig. S1).

#### Borderline results

Epitope, Euroimmun, and Wantai package inserts specified optical density ratios for definition of borderline results. One out of 178 (0.6%) positive samples tested borderline in Wantai and 8/220 (3.6%) positive samples gave a borderline result in Euroimmun. Additionally, 5/278 (1.8%) negative samples tested borderline in Euroimmun. In contrast, 11/220 (5%) of positive samples and 42/278 (15.1%) of negative samples gave a borderline result in Epitope.

#### Specificity

The specificities were > 99% for all platform-based assays except for Epitope for which the specificity was 68.7% (95% CI 62.9–74.1) (Table 2).

**Table 2:** Specificity and sensitivity of six platform-based SARS-CoV-2 antibody tests.

Platform-based antibody test	Specificity			Overall sensitivity*			Sensitivity 1–21 days post symptom onset			Sensitivity ≥ 22 days post symptom onset		
	N	%	95% CI	N	%	95% CI	N	%	95% CI	N	%	95% CI
Abbott	115	100	96.8–100	104	75.0	65.6–83.0	32	62.5	43.7–78.9	65	81.5	70.0–90.1
Epitope	278	68.7	62.9–74.1	220	71.8	65.4–77.7	72	66.7	54.6–77.3	129	75.2	66.8–82.4
Euroimmun	278	99.3	97.4–99.9	220	71.8	65.4–77.7	72	44.4	32.7–56.6	129	87.6	80.6–92.7
In-house RV	278	99.3	97.4–99.9	220	87.7	82.6–91.8	72	68.1	56.0–78.6	129	99.2	95.8–100
Mabtech	278	100	98.7–100	217	79.3	73.3–84.5	72	62.5	50.3–73.6	126	88.1	81.1–93.2
Wantai	260	99.6	97.9–100	178	88.8	83.2–93.0	65	78.5	66.5–87.7	94	96.8	91.0–99.3

*CI* confidence interval.

*Independent of time between onset of COVID-19 symptoms and sampling.

Included among the negative samples were samples collected from patients with other infectious diseases (Supplementary Text). One sample (OC43 and HKU1 co-infection) tested false-positive using Epitope, while no false-positive results to seasonal coronaviruses were observed in Euroimmun, in-house RV, or Mabtech. Due to limited sample volumes, Abbott and Wantai were not evaluated using these samples. False-positive results were also observed for Epitope when samples from patients with other viral infections, as well as bacterial and parasitic infections, were tested (Table S4). One sample from a patient positive for tularemia-specific IgM and IgG antibodies gave a false-positive result in Euroimmun. No false-positive results were observed when samples from patients with other microbial infections were tested in Abbott, in-house RV, Mabtech, and Wantai (Table S4).

#### Sensitivity

The overall sensitivity was determined by using samples from laboratory confirmed COVID-19 patients, independent of the time elapsed between sampling and symptom onset, and ranged from 72 to 89% depending on the platform-based assay. The highest overall sensitivities were observed for in-house RV and Wantai; 87.7% (95% CI 82.6–91.8) and 88.8% (95% CI 83.2–93.0), respectively (Table 2). Given that not all COVID-19 patients develop IgG antibodies before the third week of illness^5,9^, we analyzed the sensitivity for the platform-based assays by testing samples grouped by time following symptom onset. All assays but Euroimmun had > 60% sensitivity for samples collected 1–21 days post symptom onset (Table 2). When analyzing samples collected ≥ 22 days post symptom onset, the sensitivity was 99.2% (95% CI 95.8–100) for in-house RV and 96.8% (95% CI 91.0–99.3) for Wantai, while the sensitivities of the other platform-based assays ranged from 75 to 88% (Table 2).

#### Performance using samples with low IgG antibody titers

The lower sensitivity observed for platform-based assays during the first 3 weeks after symptom onset might be explained by a low seroconversion rate, low sensitivity of the assays in patients with weak antibody responses, or both. Indeed, a higher proportion (39%) of the samples in the earlier time-interval (1–21 days post symptom onset) had IFA titers ≤ 80 compared to samples in the later time-interval (22%) (Fig. S2). To investigate the effect of variations in IgG antibody titers on assay performance, we evaluated the proportion of positive tests per IFA titer (Fig. 2B). All assays detected > 80% of samples with a titer of ≥ 320. However, only in-house RV and Wantai detected > 80% of samples with a titer of 80, with Wantai detecting 100% of samples with an IFA titer of ≥ 80 (Fig. 2B).

#### Performance at early time-points after disease onset

**Figure 3:**
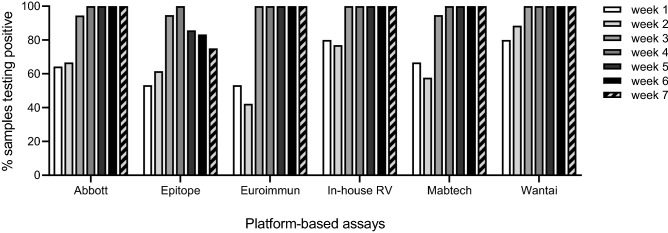
Performance of platform-based antibody tests using consecutively collected samples from 38 COVID-19 patients. The samples (n = 86) were collected during week 1–7 after onset of symptom. Weeks 1–7 are represented by 15, 26, 19, 9, 7, 6, and 4 samples, respectively. Due to a limitation in sample volumes, Abbott was evaluated using a subset of the samples (n = 78). Samples with a borderline outcome were considered negative.

Having a short window period from onset of symptom to assay positivity is a valuable assay performance characteristic. Here, the platform-based assays were assessed using consecutive samples from 38 patients. In-house RV and Wantai most frequently detected the first sample from each patient, collected during week 1 or 2 after onset of symptoms (Figs. 3, S3). This corresponded well with the ability of the assays to detect samples with low IFA titer (Table 2, Fig. 2B). By week 3 after onset of symptoms, Euroimmun, in-house RV, and Wantai detected all samples (Figs. 3, S3).

## Discussion

Antibody tests can help define prior SARS-CoV-2 infections in populations, and may in the future allow for monitoring of responses to vaccination. In addition, antibody tests can aid in the diagnosis of COVID-19 patients when NAATs are negative despite clinical suspicion. For most of these applications, the use of antibody tests with high sensitivity and specificity is critical^11^. Here, we evaluated the performances of eleven antibody tests that are based on different assay formats and SARS-CoV-2 antigens.

The assessed RDTs can detect both IgG and IgM, providing a possibility not only to identify recent and past infections but also to define a likely time-period since infection^24^. However, as heat treatment has been suggested to affect the levels of anti-SARS-CoV-2 antibodies, primarily the level of IgM antibodies^25,26^, the IgM-tests are not assessed here. We determined the IFA IgG titer for all positive samples in our reference set and all positive samples used in the evaluation of RDTs had an IgG titer of at least 80. Despite this, the sensitivity was generally low for all evaluated RDTs and many had difficulties detecting IgG titers in the lower range. Similarly, only two platform-based tests, in-house RV and Wantai, detecting IgG antibodies directed towards the S antigen and total antibodies directed towards RBD, respectively, showed satisfactory performances using samples with low IgG titers. This was reflected on the assays’ sensitivities, with a majority of platform-based assays having low sensitivity in our evaluation. A recent study shows that Abbott has lower sensitivity than other comparable antibody tests^27^, while the reported sensitivities for Epitope and Euroimmun vary depending on the study^28–30^. Both N and S antigen-based antibody detection assays have been reported to have high sensitivity^31^. With the exception of Euroimmun, the platform-based assays with S-based antigens had higher sensitivity compared to Abbott and Epitope, which are based on N protein. While the demand for sensitive SARS-CoV-2 antibody detection methods will remain, the antigens of choice may vary depending on the context such as the selection of available vaccines and the vaccination status of the population.

Our reference set mainly consisted of negative samples collected from patients seeking healthcare for non-communicable diseases and patients infected with other pathogens so as to reflect diagnostic challenges. By using this reference set, high specificity was observed for all antibody tests except Autobio RDT and Epitope ELISA. This is in line with previous reports showing generally high RDT specificity^32,33^ and > 95% specificity for Abbott, Euroimmun, and Wantai^6,29,30,34–40^, while the reported specificities for Epitope have been lower^28,38^.

To properly use serological testing, it is important to understand the limitations of antibody tests in the context in which their use is intended. Few of the SARS-CoV-2 antibody tests performed well when evaluated against samples with low IFA IgG antibody titers. Based on current knowledge, this scenario is to be expected during the first weeks of infection with SARS-CoV-2 and possibly also in the late-convalescence-phase, in asymptomatic carriers, and in immunocompromised persons. Thus, this is an important factor to take into consideration when choosing antibody tests.

In conclusion, the evaluated platform-based tests showed improved sensitivity compared to the RDTs but only two performed well when evaluated against low-titer samples. The specificity, however, was equivalently high independent of assay format.

## Supplementary Information

Supplementary Information.

## References

[1] Wiersinga WJ, Rhodes A, Cheng AC, Peacock SJ, Prescott HC (2020). Pathophysiology, transmission, diagnosis, and treatment of coronavirus disease 2019 (COVID-19): A review. JAMA.

[2] Udugama B (2020). Diagnosing COVID-19: The disease and tools for detection. ACS Nano.

[3] Kucirka LM, Lauer SA, Laeyendecker O, Boon D, Lessler J (2020). Variation in false-negative rate of reverse transcriptase polymerase chain reaction-based SARS-CoV-2 tests by time since exposure. Ann. Intern. Med..

[4] Xiang F (2020). Antibody detection and dynamic characteristics in patients with COVID-19. Clin. Infect. Dis..

[5] Long QX (2020). Antibody responses to SARS-CoV-2 in patients with COVID-19. Nat. Med..

[6] Zhao J (2020). Antibody responses to SARS-CoV-2 in patients of novel coronavirus disease 2019. Clin. Infect. Dis..

[7] Guo L (2020). Profiling early humoral response to diagnose novel coronavirus disease (COVID-19). Clin. Infect. Dis..

[8] Plebani M, Padoan A, Negrini D, Carpinteri B, Sciacovelli L (2020). Diagnostic performances and thresholds: The key to harmonization in serological SARS-CoV-2 assays?. Clin. Chim. Acta.

[9] Fafi-Kremer S (2020). Serologic responses to SARS-CoV-2 infection among hospital staff with mild disease in eastern France. EBioMedicine.

[10] Burbelo PD (2020). Sensitivity in detection of antibodies to nucleocapsid and spike proteins of severe acute respiratory syndrome coronavirus 2 in patients with coronavirus disease 2019. J. Infect. Dis..

[11] Krammer F, Simon V (2020). Serology assays to manage COVID-19. Science.

[12] Rongqing Z (2020). Early detection of severe acute respiratory syndrome coronavirus 2 antibodies as a serologic marker of infection in patients with coronavirus disease 2019. Clin. Infect. Dis..

[13] Amanat F (2020). A serological assay to detect SARS-CoV-2 seroconversion in humans. Nat. Med..

[14] Long QX (2020). Clinical and immunological assessment of asymptomatic SARS-CoV-2 infections. Nat. Med..

[15] Lee YL (2020). Dynamics of anti-SARS-Cov-2 IgM and IgG antibodies among COVID-19 patients. J. Infect..

[16] Zhang F (2020). Adaptive immune responses to SARS-CoV-2 infection in severe versus mild individuals. Signal. Transduct. Target Ther..

[17] Du Z, Zhu F, Guo F, Yang B, Wang T (2020). Detection of antibodies against SARS-CoV-2 in patients with COVID-19. J. Med. Virol..

[18] Liu A (2020). Disappearance of antibodies to SARS-CoV-2 in a COVID-19 patient after recovery. Clin. Microbiol. Infect..

[19] Chen Y (2020). A comprehensive, longitudinal analysis of humoral responses specific to four recombinant antigens of SARS-CoV-2 in severe and non-severe COVID-19 patients. PLoS Pathog..

[20] Ibarrondo FJ (2020). Rapid decay of anti-SARS-CoV-2 antibodies in persons with mild COVID-19. N. Engl. J. Med..

[21] Kampf G, Voss A, Scheithauer S (2020). Inactivation of coronaviruses by heat. J. Hosp. Infect..

[22] Varnaite R (2020). Expansion of SARS-CoV-2-specific antibody-secreting cells and generation of neutralizing antibodies in hospitalized COVID-19 patients. J. Immunol..

[23] Rosendal E (2020). Detection of asymptomatic SARS-CoV-2 exposed individuals by a sensitive S-based ELISA. MedRxiv..

[24] Theel ES (2020). The role of antibody testing for SARS-CoV-2: Is there one?. J. Clin. Microbiol..

[25] Hu X (2020). Heat inactivation of serum interferes with the immunoanalysis of antibodies to SARS-CoV-2. J. Clin. Lab. Anal..

[26] Hu X (2020). Impact of heat-inactivation on the detection of SARS-CoV-2 IgM and IgG antibody by ELISA. Clin. Chim. Acta.

[27] National SARS-CoV-2 Serology Assay Evaluation Group (2020). Performance characteristics of five immunoassays for SARS-CoV-2: A head-to-head benchmark comparison. Lancet Infect. Dis..

[28] Whitman JD (2020). Evaluation of SARS-CoV-2 serology assays reveals a range of test performance. Nat. Biotechnol..

[29] Jääskeläinen AJ (2020). Performance of six SARS-CoV-2 immunoassays in comparison with microneutralisation. J. Clin. Virol..

[30] Beavis KG (2020). Evaluation of the EUROIMMUN anti-SARS-CoV-2 ELISA assay for detection of IgA and IgG antibodies. J. Clin. Virol..

[31] Li D, Li J (2020). Immunologic testing for SARS-CoV-2 infection from the antigen perspective. J. Clin. Microbiol..

[32] Norwegian Organization for Quality Improvement of Laboratory Examinations (Noklus). Evaluation of 17 rapid tests for detection of antibodies against SARS-CoV-2. (2020) (accessed 11 October 2020); https://www.noklus.no/aktuelt/2020/april/rapport-etter-utproving-av-hurtigtester.

[33] Bond K (2020). Evaluation of serological tests for SARS-CoV-2: Implications for serology testing in a low-prevalence setting. J. Infect. Dis..

[34] Batra R (2020). A comparative evaluation between the Abbott Panbio COVID-19 IgG/IgM rapid test device and Abbott Architect SARS CoV-2 IgG assay. J. Clin. Virol..

[35] Charpentier C (2020). Performance evaluation of two SARS-CoV-2 IgG/IgM rapid tests (Covid-Presto and NG-Test) and one IgG automated immunoassay (Abbott). J. Clin. Virol..

[36] Manalac J (2020). Evaluation of Abbott Anti-SARS-CoV-2 CMIA IgG and Euroimmun ELISA IgG/IgA assays in a clinical lab. Clin. Chim. Acta.

[37] Bryan A (2020). Performance characteristics of the Abbott Architect SARS-CoV-2 IgG assay and seroprevalence in Boise, Idaho. J. Clin. Microbiol..

[38] van Kessel CHG (2020). An evaluation of COVID-19 serological assays informs future diagnostics and exposure assessment. Nat. Commun..

[39] Kruttgen A (2020). Comparison of four new commercial serologic assays for determination of SARS-CoV-2 IgG. J. Clin. Virol..

[40] Meyer B (2020). Validation of a commercially available SARS-CoV-2 serological immunoassay. Clin. Microbiol. Infect..

