# Supplementary material for: Evaluation of 11 SARS-CoV-2 antibody tests by using samples from patients with defined IgG antibody titers
Source: Sci Rep. 2021 Apr 7;11:7614. doi: 10.1038/s41598-021-87289-6 (PMC8027209; doi:10.1038/s41598-021-87289-6)
Supplement: Supplementary file 1 — Supplementary Information. [file 41598_2021_87289_MOESM1_ESM.pdf]

Evaluation of 11 SARS-CoV-2 antibody tests by using samples from patients with defined IgG antibody titers

Nina Lagerqvist[1#], Kimia T. Maleki[1,2], Jenny Verner-Carlsson[1], Mikaela Olausson[1], Joakim Dillner[3], Julia Wigren Byström[4], Tor Monsen[4], Mattias Forsell[4], Jenny Eriksson[5], Gordana Bogdanovic[6], Sandra Muschiol[6], Joel Ljunggren[7], Johanna Repo[7], Torbjörn Kjerstadius[8], Shaman Muradrasoli[1], Mia Brytting[1], Åsa Szekely Björndal[1], Thomas Åkerlund[1], Charlotta Nilsson[1,3], and Jonas Klingström[1,2]

[1]Department of Microbiology, Public Health Agency of Sweden, Solna, Sweden

[2]Department of Medicine Huddinge, Karolinska Institutet, Stockholm, Sweden

[3]Department of Laboratory Medicine, Karolinska Institutet, Stockholm, Sweden

[4]Department of Clinical Microbiology, Umeå University, Umeå, Sweden

[5]Region Västmanland, Västerås, Sweden

[6]Karolinska University Hospital, Stockholm, Sweden

[7]Region Västernorrland, County Hospital of Västernorrland, Sundsvall, Sweden

[8]Region Värmland, Centralsjukhuset, Karlstad, Sweden

[#]Address correspondence to Nina Lagerqvist, nina.lagerqvist@folkhalsomyndigheten.se

Supplementary Information

Text

Bacterial diseases included leptospirosis, tularemia, brucellosis, Q fever, Mediterranean spotted fever, melioidosis, and scrub typhus. Parasitic diseases included malaria and schistosomiasis. Viral diseases included infections with common human coronaviruses, dengue virus, zika virus, West Nile virus, Japanese encephalitis virus, chikungunya virus, and hantavirus causing hemorrhagic fever with renal syndrome.

Table S1. Assay characteristics.

Acronym	Test	Manufacturer	Format	Lot no.	Antibody	Target	Specimens
Acro	2019-nCoV IgG/IgM Rapid Test	Acro Biotech Inc., Rancho Cucamonga, CA, USA	RDT	NCP20030226, NCP20030143, NCP20030100	IgM and IgG	NA*	Whole blood, serum, plasma
Autobio	Anti-SARS-CoV-2 Rapid Test	Autobio Diagnostics Co. Ltd, Zhengzhou, China	RDT	21C22-J01	IgM and IgG	spike	Serum, plasma
Healgen	COVID-19 IgG/IgM Rapid Test	Healgen Scientific Limited Liability Company, Houston, TX, USA / Zhejiang Orient Gene Biotech Co. Ltd, Zhejiang, China	RDT	2003242, 2003310	IgM and IgG	spike S1	Whole blood, serum, plasma
Nadal	NADAL COVID-19 IgG/IgM Test	Nal von Minden GmbH, Moers, Germany	RDT	COV20030017	IgM and IgG	NA*	Whole blood, serum, plasma
OnSite	OnSite COVID-19 IgG/IgM Rapid Test	CTK Biotech Inc., Poway, CA, USA	RDT	RD1625	IgM and IgG	spike S1	Whole blood, serum, plasma
Abbott	Architect SARS-CoV-2 IgG	Abbott, Chicago, IL, USA	CMIA	18510FN00	IgG	N	Serum, plasma
Epitope	EDI Novel coronavirus COVID-19 IgG ELISA	Epitope Diagnostics Inc., San Diego, CA, USA	ELISA	P738C, P679U	IgG	N	Serum
Euroimmun	Anti-SARS-CoV-2 ELISA (IgG)	Euroimmun, Lübeck, Germany	ELISA	E200414BG, E200416AE, E200420AW, E200428BI, E200407AV, E200429AG	IgG	spike S1	Serum, plasma
In-house RV	In-house Region Västerbotten	(23)	ELISA	NA	IgG	spike	Heat-inactivated plasma or serum
Mabtech	Mabtech SARS-CoV-2 Spike S1-RBD Ig Bridge ELISA	MabTech AB, Stockholm, Sweden	ELISA	NA	Ig	spike S1-RBD	Serum, plasma
Wantai	Wantai SARS-CoV-2 Ab ELISA	Beijing Wantai Biological Pharmacy Enterprise Co., Ltd., Beijing, China	ELISA	NCOA20200401	Ig	RBD	Serum, plasma

RDT, rapid diagnostic test; CMIA, chemiluminescent microparticle immunoassay; N, nucleocapsid protein; NA, not available; RBD, receptor-binding domain.

*not specified in the manufacturer's instructions

Table S2. Number of inconclusive rapid diagnostic test IgG-results.

Rapid diagnostic test	Positive samples (N = 87)		Negative samples (N = 96)	
	n	%	n	%
Acro	7	8.0	1	1.0
Autobio	11	12.6	1	1.0
Healgen	7	8.0	0	
Nadal	8	9.2	0	
OnSite	3	3.4	1	1.0

Table S3. Rapid diagnostic test IgG-results reported by two laboratory technicians.

		**Operator 2**		
		Negative	Positive	Inconclusive
**Operator 1**	Negative	576	0	2
	Positive	0	296	5
	Inconclusive	3	0	33

Table S4. Samples testing false positive in platform-based antibody tests.

**Platform-based assay**	**Healthy donors**	**Persons seeking medical care**	**Patients with infectious diseases**		
			Bacteria	Virus	Parasite
	n/N	n/N	n/N	n/N	n/N
Abbott	0/19	0/79	0/3	0/14†	NA
Epitope*	7/35	70/164	5/32	4/40	1/7
Euroimmun*	0/35	1/164	1/32	0/40	0/7
In-house RV	0/35	2/164	0/32	0/40	0/7
Mabtech	0/35	0/164	0/32	0/40	0/7
Wantai*	0/35	1/164	0/32	0/24†	0/5

NA, not analyzed

*A borderline outcome was considered a negative result.

†Samples from patients infected with seasonal coronaviruses were not available due to limitations in sample volumes.

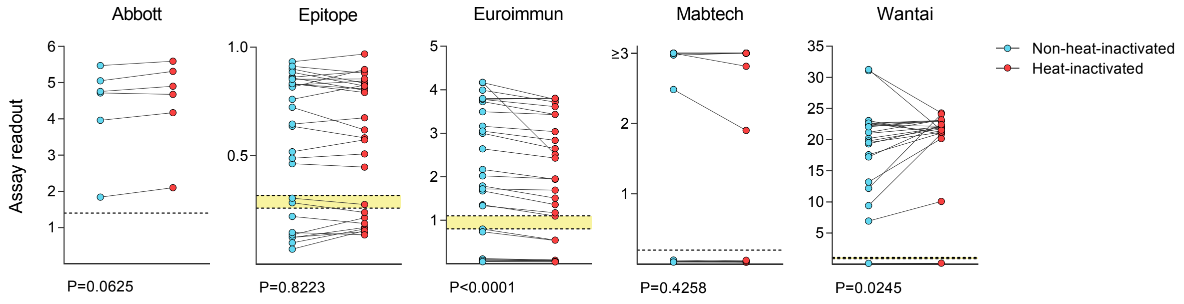

Figure S1. Effect of sample heat-inactivation on assay readout. Epitope, Euroimmun, Mabtech, and Wantai were evaluated using non-heat-inactivated (white dots) and heat-inactivated (grey dots) aliquots of 6 negative and 21 positive samples and Abbott using 6 positive samples. Dotted lines correspond to the cutoff and the yellow area shows the borderline interval specified by the manufacturer. Differences in readout-values between non-heat-inactivated and heat-inactivated sample aliquots were analyzed using Wilcoxon matched-pairs signed rank test.

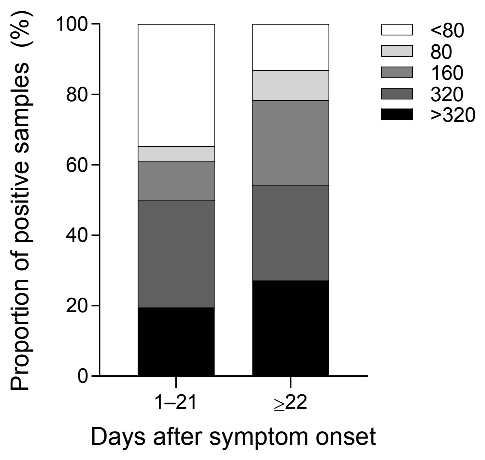

Figure S2. Distribution of IgG titers among samples collected 1–21 and ≥22 days after symptom onset. The IgG titer was determined by using an in-house immunofluorescence assay. The two time-intervals, 1–21 and ≥22 days after symptom onset, are represented by 72 and 129 samples, respectively.

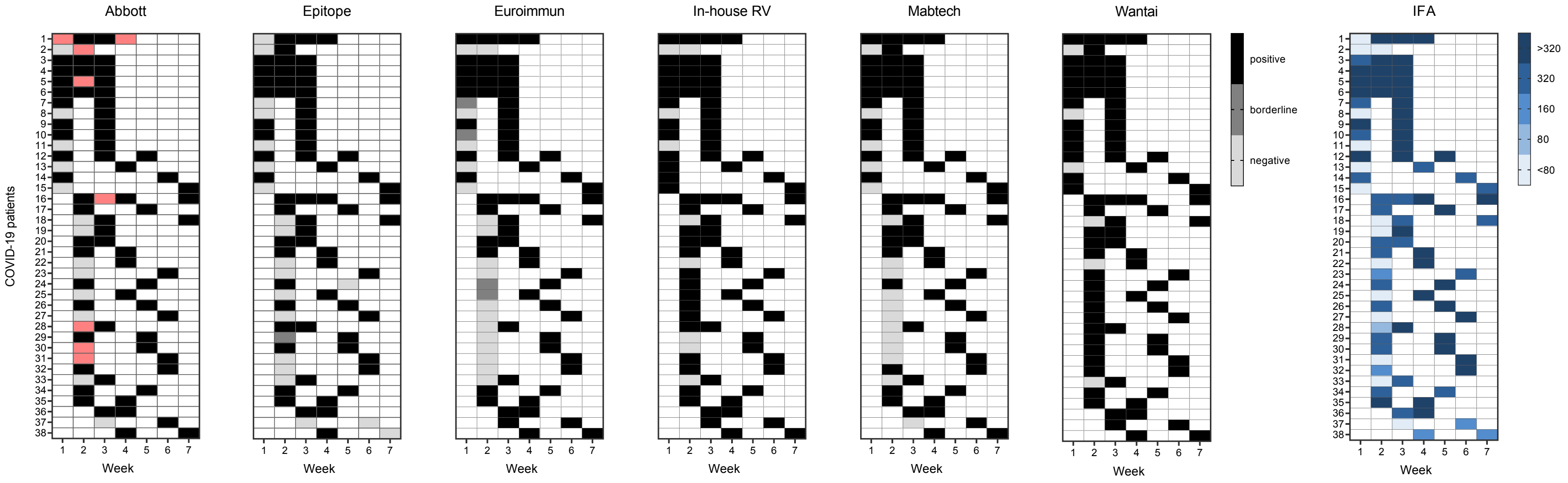

Figure S3. Performance of platform-based antibody tests using consecutively collected samples from 38 COVID-19 patients. The samples were collected during week 1-7 after onset of symptom and were titrated and analyzed for anti-SARS-CoV-2 IgG antibodies using an in-house immunofluorescence assay (IFA). Each row represents one patient. Due to a limitation in sample volumes, Abbott was evaluated using a subset of the samples and the red cells represent missing sample.
